# Effects of storage on volatile organic components and physiological properties of different storage-tolerant rice varieties

**DOI:** 10.1016/j.fochx.2024.102134

**Published:** 2024-12-27

**Authors:** Dawei Zhu, Xin Zheng, Huiyin Dong, Xingquan Liu, Xianqiao Hu, Mingxue Chen, Xin Liu, Yafang Shao

**Affiliations:** a Rice Product Quality Supervision and Inspection Center, Ministry of Agriculture and Rural Affairs, China National Rice Research Institute, Hangzhou 310006, China; b Seed Management Station of Zhejiang Province, Hangzhou 310006, China; c Department of Food Science, Zhejiang A & F University, Hangzhou, Zhejiang 311300, China

**Keywords:** Rice, Storage, Volatile organic components, GC-IMS, Physiological properties

## Abstract

The effects of storage on rice flavor among different rice varieties have not been well studied. To address this gap, we analyzed volatile organic components (VOCs) identified by gas chromatography-ion mobility spectrometry (GC-IMS) and related physicochemical properties of different storage-tolerant rice varieties during storage. The results showed that VOCs of four rice varieties significantly changed after 6 months of storage; OPLS-DA analysis classified the four rice varieties into two groups. There were fewer (N81 and JH1) and more significant changes (N84 and ZJ96) after storage, and the hexanal and 2-pentylfuran were considered the key VOCs for flavor changes during storage. Lipoxygenase (LOX) activity first increased and then decreased, while antioxidant activities decreased during storage. Under these conditions, oleic and linoleic acids were hydrolyzed. These results provide a better understanding of rice flavor changes after storage between different storable rice varieties.

## Highlights

• Different storage tolerant *japonica* rice varieties were studied.
• 2-Pentylfuran and hexanal are the key volatile organic components during storage.
• Storage tolerant *japonica* rice varieties had a high SOD and POD activity.

## 1 Introduction

Rice is the most important staple crop in the world and feeds more than 60 % of the world's population. Though rice production is seasonal, it is consumed all year round, which means it will be stored for a period before consumption. Rice can be stored in multiple forms, such as brown rice, milled, or paddy rice, among which paddy rice is the predominant storage strategy (Yuan et al., 2019). Paddy rice is dormant during storage, but its internal physiological and metabolic activities continue, which induces physicochemical and biochemical reactions, including starch hydrolysis and lipids oxidation (Gao et al., 2016). In China, it has been reported that more than 3 % of rice is lost during storage every year (Nie & Peng, 2017). Especially as global temperature rises, there is a greater risk to rice growth and storage (Huang et al., 2023). Therefore, clarifying the degradation rule of rice quality during storage is very important. Our previous study found that changes in the molecular structure of starch during storage were the driving force for the degradation of rice flour, leading to gelatinization and degrading the texture of cooked rice (Zhu, Li, et al., 2023).

In addition to the textural quality, rice flavor is an essential characteristic of rice eating quality (Dias et al., 2019; Verma et al., 2012). A major change during storage is the oxidation of lipids, which is an important factor affecting cooked rice's aroma. Lipids are hydrolyzed by lipase into glycerol and fatty acids during storage, and these products continue to oxidize and decompose into ketones, aldehydes, and alcohols through reactions with enzymes (Jungtheerapanich et al., 2017; Liu, Liu, et al., 2021). The volatile components produced from these reactions cause an unpleasant smell in cooked rice after storage, affecting rice eating and cooking quality. The substantial effect on such an important crop justifies previous and ongoing research on volatile components in rice during storage. Shi et al. (2021) studied the effects of high temperature on the flavor components of *japonica* rice during storage and found that the increase of the main components of hydrocarbons and ketones during storage was influenced by accumulated temperature. The rise in aldehydes, ketones, and furans under high-temperature storage was the main reason for the flavor change of fragrant rice, according to a study by Zhao, Yousaf, et al. (2020). Hu et al. (2023) found different volatile compounds among *japonica* and indica rice during storage, and that increased aldehydes, ketones, and furans negatively impacted rice aroma during storage. In short, the volatile compounds of rice significantly change during storage, which can produce unpleasant flavor and affect rice eating quality.

Although there has been research on the volatile components of rice varieties after storage, they have mainly focused on the effects of storage conditions (such as temperature or humidity) or milling status (paddy rice, brown rice, or milled rice) on volatile compounds after storage. Few studies investigated the changes in volatile components between different genotype rice varieties during storage. Previous studies have demonstrated that changes in rice quality were not only related to storage conditions but also influenced by the genotype of the rice variety, though the effect of rice genotype has not been characterized (Yuan et al., 2019; Zhu, Shao, et al., 2023). In this study, two groups of rice varieties with different storage tolerances were selected, and the flavor fingerprints during storage were analyzed by GC-IMS, as well as LOX activity, fatty acid value, and fatty acid components. These results provide valuable insight for research on rice flavor changes during storage.

## 2 Materials and methods

### 2.1 Materials and storage condition

Four *japonica* rice varieties with different storage-tolerant were used in this study; there were fewer storage-tolerant varieties Ningjing 84 (N84) and Zhejing 96 (ZJ96), storage-tolerant varieties Ningjing 81 (N81) and Jiahua 1 (JH1). We defined storage tolerance based on the variation in rice eating quality and fatty acid values before and after storage. The information of four rice varieties is in Table S1. These varieties were harvested from the experimental field in 2022. After removing the impurities, the moisture contents of rice samples were controlled at 13–14 %, then packaged in nylon net bags and stored in an artificial climate chamber (Memmert, HPP260, Germany) (35 °C, 75 % humidity). Samples were collected at the beginning of the experiment (CK), and after 2, 4, and 6 months of storage.

### 2.2 Determination of fatty acid value (FAV) and malondialdehyde (MDA)

FAV was determined according to the protocol of the American Association of Cereal Chemists American Association of Cereal Chemists (AACC) (1976). As previously described, 10.00 g of the dry rice sample was added to 50.0 mL of anhydrous ethanol, and shaken for 10 min at a frequency of 100 shakes per minute. 25.0 mL of filtrate was taken, combined with 50 mL of distilled water lacking carbon dioxide, and then 3–4 drops of phenolphthalein indicator were added. FAVs are expressed as the amount of potassium hydroxide required to neutralize free fatty acids in 100 g of dry sample material with units of mg of free fatty acid /100 g dry sample material (mg KOH/ 100 g).

The MDA content was analyzed according to Spitz and Oberley (1989) with minor modifications. 0.1 g of dry rice sample was added to 1 mL of phosphate buffer and then centrifuged at 8000 ×*g* for 10 min at room temperature. 100 μL of the supernatant was collected, added to 300 μL of thiobarbital acid solution, mixed well, and then incubated in a water bath at 92 °C for 30 min. After cooling, the sample was centrifuged at 8000 ×*g*, 25 °C for 10 min. 200 μL of the supernatant was collected in a 96-well plate, and absorbance at 532 nm and 600 nm were measured by spectrophotometer.

### 2.3 Determination of enzyme activity

The activity of superoxide dismutase (SOD, EC 1.15.1.1) was determined according to Ukeda et al. (1999) with minor modifications. A 0.1 g dry rice sample was mixed with 1 mL of phosphate buffer, homogenized in an ice bath manually, and centrifuged at 10000 ×*g*, 4 °C for 10 min. The sample was mixed with assay solution and blank solution in separate wells of a 96-well plate and incubated for 30 min at room temperature; at this point, the absorbance at 450 nm was measured. SOD activity at 50 % inhibition in the reaction system was defined as one unit of enzyme activity (U/mL).

Peroxidase activity (POD, EC 1.11.1.7) was determined according to Reuveni et al. (1992) with minor modifications. A sample of 0.1 g was mixed with 1 mL of phosphate buffer, homogenized in an ice bath, and centrifuged at 10000 ×*g*, 4 °C for 10 min. Then,10 μL of the supernatant and 190 μL of the 0.05 mol/L guaiacol solution were added to a 96-well plate, mixed well, and the absorbance at 470 nm was measured at 10 s and 70 s. A change in absorbance of 0.5 Au at 470 nm per minute for a gram of tissue per milliliter of the reaction system was defined as a unit of enzyme activity (U/g).

The lipoxygenase (LOX) activity was determined according to Yalcin and Basman (2015) with minor modifications. 0.1 g of dry rice sample was mixed with 1 mL of phosphate buffer, homogenized in an ice bath, and centrifuged at 10000 ×*g*, 4 °C for 10 min, and the supernatant was collected for measurement. The assay and blank solutions were prepared, and 200 μL of each was transferred to a 96-well plate. The absorbance was measured at 234 nm after a 5-min incubation at room temperature (25 °C). A change in absorbance value of 0.01 Au at 234 nm per minute for a gram of tissue per milliliter of the reaction system at 25 °C was defined as a unit of enzyme activity (U/g).

### 2.4 Determination of sucrose content

To measure the sucrose content, we used the protocol published by Li et al. (2017) with minor modifications. Sucrose was detected using a high-performance liquid chromatography (HPLC) instrument (2695, Waters, USA) equipped with an Ultimate HILIC-NH2 column (250 mm × 4.6 mm, 5 μm). 0.2 g of rice flour and 1.0 mL of 20 % ethanol were added, and the mixture was ground. After grinding the sample, it was sonicated for 1 h and extracted overnight; then, the supernatant was collected by centrifugation and filtered. The HPLC flow rate was 1.0 mL/min, the injection volume was 10 μL, and the mobile phase was 80 % acetonitrile aqueous solution. Sucrose standard curve samples were generated in-house using sucrose dissolved in water across various concentrations. The peak areas of each standard solution were measured sequentially using the described chromatographic conditions. The standard curve, linear range, and correlation coefficient of sucrose were obtained by taking the peak area as the vertical coordinate and the concentration as the horizontal coordinate.

### 2.5 Determination of fatty acids components

The fatty acids were separated and evaluated by gas chromatography with a flame ionization detector (Agilent 6890, Agilent Technologies Co., USA) according to Hu et al. (2023). 500 mg of dry rice sample was added to 1 mL of petroleum ether-ether mixture, then 1 mL of potassium hydroxide-methanol solution was added as the methyl esterification reagent. The mixture was vortexed and then incubated for 1 h at room temperature. After shaking again, 1 mL of deionized water was added, and the mixture was left to stand for 30 min at room temperature, then centrifuged at 4000 ×*g* for 2 min, before the supernatant was collected.

The chromatographic conditions were as follows: using a DB-225MS column (30 m × 0.25 mm × 0.25 μm) with the injection port heated to 250 °C, the column was held at 50 °C for 1 min, and then increased to 200 °C at a rate of 5 °C/min, and then increased again to 230 °C at a rate of 2 °C/min and held for 10 min. The carrier gas was helium (99.999 %) with a 3 mL/min flow rate. Samples were injected with a split ratio of 10:1 and an injection volume of 1 μL. For GC–MS, the transfer line temperature was 250 °C. For mass spectrometry, the ion source temperature was 230 °C, the quadrupole temperature was 150 °C. the ionization mode was EI, the solvent delay time was 3 min, the data acquisition mode was full scan, and the mass-to-charge ratio (*m*/*z*) was 35–800. The peak areas of each standard solution were detected sequentially according to the above chromatographic conditions, and the standard curves, linear ranges, and correlation coefficients of the fatty acids were obtained by taking the peak area as the vertical coordinate and the concentrations as the horizontal coordinates.

### 2.6 Volatile organic compounds analysis based on GC-IMS

Rice Volatile Organic Compounds were analyzed by using HS-GC-IMS consisting of a 60-position headspace auto-sampler (PAL RSI, CTC Analytics AG, Zwingen, Switzerland), an Agilent 490 Micro GC (Agilent Technologies, USA), and a FlavourSpec® advanced IMS (G.A.S. mbH, Germany) according to Zhang et al. (2020) with minor modifications. 3 g of rice was weighed into a 20 mL headspace sample bottle, sealed airtight, and assayed. With the headspace injection needle temperature at 85 °C, the injection volume was 500 μL, and the cleaning time was 30 s. For the HS-GC-IMS, the column used was FS-SE-54-CB-1 (non-polar, 15 m × 0.53 mm × 0.5 μm). The column temperature was set to 60 °C with a run time of 25 min and carrier gas of high-purity nitrogen (purity ≥99.999 %). The flow rate was programmed as follows: maintained the initial flow rate (2.0 mL/min) for 2 min, followed by a linear increase to 10 mL/min within 10 min to 100 mL/min within 20 min, and 150 mL/min within 25 min. For the IMS, the length of the drift tube was 5 cm, the linear voltage inside the tube was 400 V/cm, the temperature of the drift tube was 45 °C, the drift gas was high-purity nitrogen (purity ≥99.999 %), the flow rate was 150 mL/min, and the temperature of the IMS detector was 45 °C.

### 2.7 Quantitative analysis of VOCs based on gas chromatography–mass spectrometry (GC–MS)

Accurately weighed 2.0 g of rice powder in a 15 mL extraction vial, added 10 μL of 2-methyl-3-heptanone (1 μg/mL, the solvent was anhydrous ethanol), and then added 10 μL of anhydrous ethanol and four series of standard solutions (Table 1), respectively. The solid phase microextraction (SPME) fiber ((DVB/CAR/PDMS, 50/30 μm, 1 cm), Anpel, Shanghai, China) was exposed to the headspace of the vial at a temperature of 90 °C for 80 min, then inserted into the injection port of GC–MS (7200, Agilent, California, USA) and desorbed at 250 °C for 5 min. The volatile compounds were separated and evaluated by using a DB-WAX column (30 m × 0.25 mm × 0.25 μm, Agilent Technologies Co.), with high-purity helium (purity >99.999 %) as carrier gas at a flow rate of 1 mL/min, and the split ratio was 10:1. The oven temperature was set at 40 °C for 5 min, increased to 150 °C at 5 °C/min, then increased to 230 °C at 10 °C/min. The mass selective detector was operated in full scan mode (*m*/*z* 40–500). The ion source was EI, with a temperature of 230 °C, and the voltage was 70 eV.

The mass spectra of hexanal and 2-pentylfuran were identified in the NIST spectral database. The standard addition working curve was plotted using the content of hexanal and 2-pentylfuran as the independent variable, and the peak area ratio of hexanal and 2-pentylfuran to 2-methyl-3-heptanone as the dependent variable (Table S2). The content of hexanal and 2-pentylfuran in the sample was calculated by extrapolation.

### 2.8 Statistical analysis

The least significant difference (LSD) test was performed for multiple comparisons (*P* < 0.05). SigmaPlot 10.0 was used to draw the graphs. The data pre-processing and OPLS-DA modeling processes were developed using SIMCA 14.1. Figures were drawn using Microsoft PowerPoint 2016.

## 3 Results and discussion

### 3.1 Changes in FAV and MDA

**Fig. 1:**
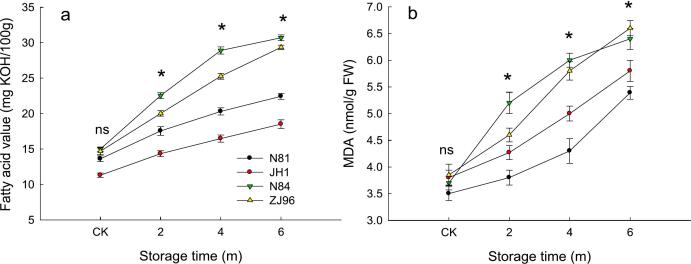
FAV (a) and MDA (b) of four rice varieties during storage. ns indicates that values of the four varieties at the same storage stage showed no significant difference; * indicates that values of the four varieties at the same storage stage showed a significant difference (*P* < 0.05).

The FAV of N81, JH1, N84, and ZJ96 increased by 64.7 %, 63.8 %, 104.8 %, and 99.9 % after 6 months of storage, respectively (Fig. 1a). The initial FAV of these four varieties showed no significant difference. After 6 months of storage, the average FAV of N84 and ZJ96 were significantly higher than that of N81 and JH1. Previous studies demonstrated that FAV increased after storage, and higher FAV in rice was observed at higher storage temperatures than in rice stored at lower temperatures (Park et al., 2012). MDA content also increased after storage (Fig. 1b). Similar to FAV, no significant difference was observed in initial MDA content. However, after 6 months of storage, the MDA content of ZJ96 was significantly higher than that of N81. Increased MDA content indicated the degree of membrane lipid peroxidation of rice (Esterbauer et al., 1991). In contrast to our result, a previous study found that MDA initially increased and then decreased after 12 months of storage (Zhang et al., 2024). The differences in results may be primarily attributed to storage conditions; the extended storage time caused the enzyme activity and FAV to decrease, reducing MDA content. Changes in FAV and MDA after storage indicated that the four varieties addressed in this research had different degrees of lipid oxidation during storage.

### 3.2 Changes in volatile organic compounds (VOCs) of rice during storage

**Fig. 2:**
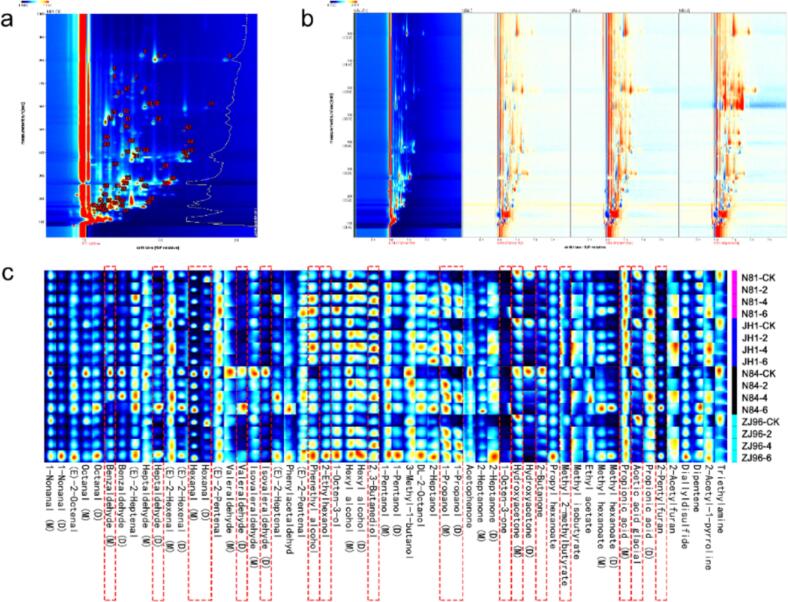
Gas phase ion migration spectrogram of N84 CK (a), difference diagram of gas phase ion migration spectrogram of N84 CK during storage (b), and fingerprinting of volatile organic compounds of four varieties during storage selected from the GC-IMS (c). The color scale from blue to red represents the signal intensity from 0 to 1.0, and the different numbers represent the peak of the identified compound based on the GC-IMS database. Each independent pixel in (c) represents a GC-IMS characteristic absorption peak. The color scale from blue to red represents the signal intensity from low to high, and black indicates background. D, dimer; M, monomer).

A total of 57 VOCs were identified in fresh and stored rice, including twenty-two aldehydes, thirteen alcohols, seven ketones, six esters, three acids, two furans, one alkene, and three other compounds (Fig. 2a, Table S3). The relative content levels of these compounds in fresh rice can be described as aldehydes > alcohols > ketones > acids (Fig. S1). To analyze the effects of storage time on VOCs of rice, we downscaled spectrograms via a differential comparison mode (Fig. 2b). Using the fresh rice samples as a nce, we subtracted the remaining three samples stored at different times from the nce, and the background color faded to white after the deduction. If the amount of VOCs in a sample is higher than the fresh rice, the substance is shown in red; otherwise, it is blue. Fig. 2c shows the fingerprints of 57 characteristic ion peaks, with the y-axis representing samples stored at different times and the x-axis representing different VOCs. After storage, the relative content of alcohols and furans increased in all four rice varieties assayed compared with other compounds (Fig. S1).

**Fig. 3:**
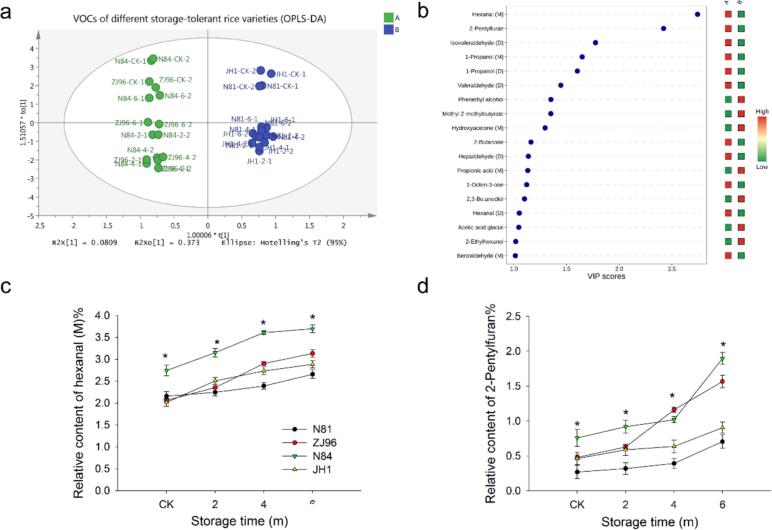
OPLS-DA model based on 57 VOCs of rice varieties during storage (a), VIP value of OPLS-DA model (b), the relative content of hexanal (M) and 2-pentylfuran of rice varieties during storage (c, d). * indicates that values of the four varieties at the same storage stage showed a significant difference (P < 0.05).

We modeled the VOCs of rice samples using orthogonal projections to latent structures-discriminant analysis, OPLS-DA (Fig. 3a), and found that the samples were generally within the 95 % confidence interval. The model of OPLS-DA was well fit, where the values of R^2^X and R^2^Y were close and the Q^2^ > 0.5 (R^2^X = 0.95, R^2^Y = 0.99, and Q^2^ = 0.943). Horizontal coordinates indicated the differences between rice varieties. It was observed that the VOCs of four rice varieties could be broadly divided into two groups: Group A was N84 and ZJ 96, and Group B was N81 and JH1, respectively. The vertical coordinates represent the differences within the group. All four varieties showed significant changes in VOCs after storage compared to fresh samples. However, compared with group A (N81 and JH1), VOCs of group B (N84 and ZJ 96) showed a greater change during storage. This means that the VOCs of the B group exhibited greater variability during storage. By comparing group A (N84 and ZJ 96) and B (N81 and JH1), VIP analysis (VIP > 1) (Fig. 3b) of the OPLS-DA model showed that the 18 volatile flavor compounds between the two groups, there were 2-pentylfuran, hexanal (M), isovaleraldehyde (D) etc. Among them, 2-pentylfuran and hexanal (M) showed the highest VIP value (2.74 and 2.42). Hexanal (M) and 2-pentylfuran after storage in four rice varieties both increased, and N84 showed a higher relative content of hexanal (M) than other rice varieties during storage (Fig. 3c, d). Different for hexanal (M), the relative content of 2-pentylfuran significantly increased at the beginning of four months of storage, and the contents of 2-pentylfuran of ZJ96 and N84 were higher than N81 and JH1. In alignment with our results, hexanal has been reported to be a potential marker molecule of lipid peroxidation in seeds during storage (Colville et al., 2012; Mira et al., 2016). Increased hexanal during storage was mainly attributed to oxidation of linoleic acid. Previous study reported that linoleic acid would be oxidized to form hydrogen peroxide during storage, which would be further degraded into hexanal, 2-octenal, and other aldehydes (Gao et al., 2021). 2-Pentylfuran, the product of lipid oxidation, including predominantly linoleic acid, is a key marker for rice aging. Which exhibits a licorice and beans odor at low concentrations and an unpleasant flavor at high concentrations (Griglione et al., 2015; Resconi et al., 2018). A previous study reported that hexanal and 2-pentylfuran had a low odor threshold (0.005 and 0.0058 mg/kg), and they were important aroma-related compounds in rice (Burdock, 2010; Giri et al., 2010). We analyzed the contents of hexanal and 2-pentylfuran in fresh rice based on GC/MS; their levels of four varieties ranged from 350.8 to 503.7 ng/g and 22.7–36.5 ng/g, respectively (Fig. S2). This suggested that higher contents of 2-pentylfuran and hexanal after storage would cause an unpleasant flavor in rice. It was noted that 18 VOCs included both monomer (M) and dimer (D) forms, which might lead to different properties even in equivalently stored rice (Zhang et al., 2022). In this study, although hexanal (M) and hexanal (D) of the four tested varieties showed the same trends during storage, hexanal (M) showed a higher VIP value than hexanal (D). It indicated that hexanal (M) might be more suitable as a marker for odor changes during storage.

### 3.3 Changes in fatty acids and sucrose contents of brown rice

**Fig. 4:**
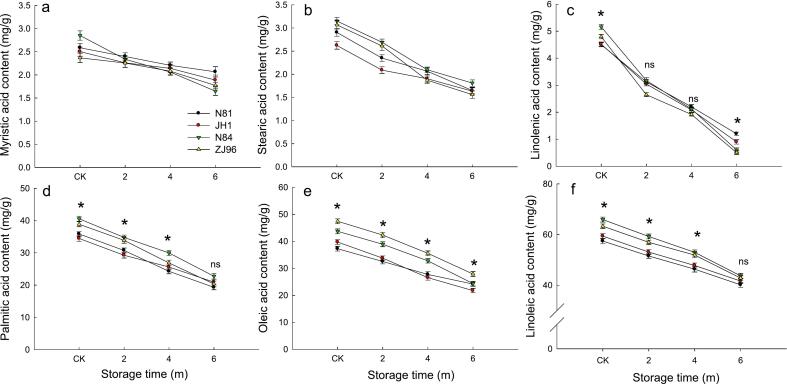
Fatty acids components of four rice varieties during storage. ns indicates that values of the four varieties at the same storage stage showed no significant difference (*P* < 0.05), * means values of the four varieties at the same storage stage showed a significant difference (*P* < 0.05).

Linoleic acid, oleic acid, palmitic acid, myristic acid, stearic acid, and linolenic acid were detected in brown rice (Fig. 4). Linoleic acid, oleic acid, and palmitic acid were the most predominant fatty acid compounds, and these fatty acid compounds significantly decreased after storage. Palmitic acid, oleic acid, and linoleic acid were the primary saturated, monounsaturated, and polyunsaturated fatty acids, respectively, in paddy rice (Liu et al., 2023). During storage, lipids would be hydrolyzed and oxidized to a series of products, including fatty acids or aldehydes and ketones (Yuan et al., 2019), and the fatty acids would be further decomposed. The unsaturated fatty acids were oxidized by lipoxygenase and then degraded into aldehydes. A decrease in linoleic acid level was likely due to being oxidized to form hydrogen peroxide during storage, which would be further degraded into hexanal, 2-octenal, and other aldehydes (Gao et al., 2021). Decreased oleic acid might be attributed to its degradation into nonanal, decanal, and other volatiles during storage (Zhao, Yousaf, et al., 2020). Interestingly, in the fresh rice, the contents of linoleic and oleic acid in less storage-tolerant varieties (ZJ96 and N84) were significantly higher than those in storage-tolerant varieties (N81 and JH1) with no significant difference after 6 months of storage. This means that the linoleic and oleic acids of two less storage-tolerant varieties degraded more than those after 6 months of storage.

**Fig. 5:**
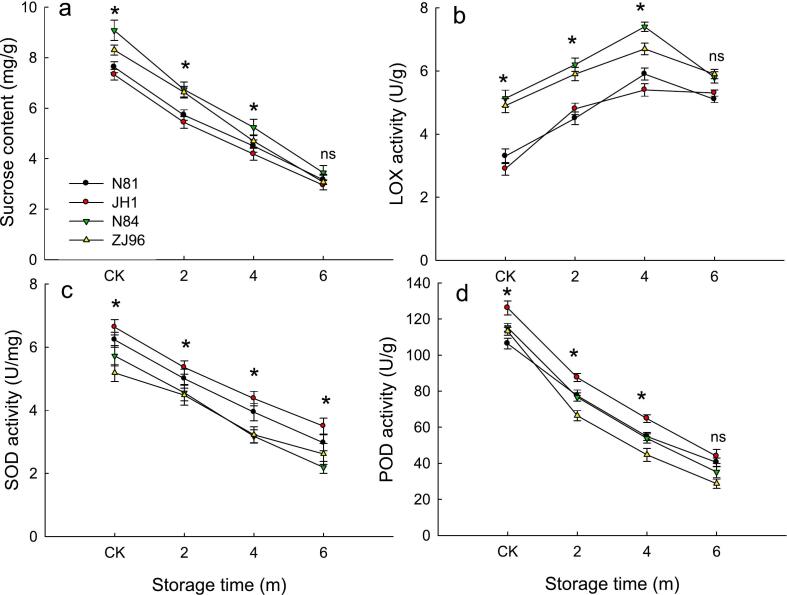
Sucrose content (a), LOX activity (b), and SOD and POD (c, d) of four rice varieties during storage. ns indicates that values of the four varieties at the same storage stage showed no significant difference (*P* < 0.05), * means values of the four varieties at the same storage stage showed a significant difference (*P* < 0.05).

Sucrose is the most abundant soluble sugar in rice, accounting for more than 80 % of the total sugar (Frank et al., 2012). After storage, we observed a decrease in the sucrose content of N81, JH1, N84, and ZJ96 by 58.5 %, 59.8 %, 62.1 %, and 63.0 %, respectively, after 6 months of storage (Fig. 5a). Significant differences were observed in fresh, 2-, and 4-month stored rice among the four rice varieties, with N84 showing the highest sucrose content. This result was aligned with prior research stating that sucrose was hydrolyzed during storage (Zhao, Xue, & Shen, 2020). Sucrose is the main factor contributing to rice's sweetness (Hu et al., 2023), which means decreased sucrose content during storage would reduce rice sweetness. Additionally, decreased sucrose content will also decrease the Maillard reaction during rice cooking which will affect rice quality. Prior studies only considered the negative effect of decreased sucrose content after storage on rice aroma quality. In addition, changes in sucrose content during storage could respond to the physicochemical activity of rice grains during storage. It has been identified that the differentially expressed genes with the most significant changes during rice storage were those in pathways related to starch and sucrose (Zhao et al., 2017). The primary mechanism causing decreased sucrose content was the downregulation of the genes encoding sucrose transferases and hydrolases. The greater the sucrose content decreased during storage, the higher the intrinsic physiological activity of rice during storage. There is a greater degree of rice oxidation, and eventually, significant changes in VOCs after storage.

### 3.4 Changes in activities of lipoxygense (LOX), superoxide dismutase (SOD), and peroxidase (POD)

LOX is an essential enzyme in the fatty acid metabolism pathway and plays a central role in rice flavor after storage. LOX activity increased in the four rice varieties during the initial 4 months of storage and decreased after 6 months (Fig. 5b). This result was similar to a previous study, which reported that LOX activity first increased and then decreased steadily with the increase in storage lengths (Liu et al., 2023). LOX could degrade unsaturated fatty acids, including oleic acid, linoleic acid, and linolenic acid (Yao et al., 2018), which could then be degraded into aldehydes, ketones, or other furan volatiles, which produce an unfavorable flavor. It has been observed that rice varieties with lower expression levels of *LOX*-3 had less stale flavor profiles when compared with rice with normal *LOX*-3 levels after storage (Suzuki et al., 1999). The electron beam irradiation could decrease the LOX activity during storage to prevent this degradation in rice quality (Liu et al., 2023). Xu et al. (2015) demonstrated that downregulating *LOX*-3 expression could reduce lipid oxidation during rice aging. This study found that the LOX activity of N84 and ZJ96 was significantly higher than that of N81 and JH1 during storage, especially in fresh rice. Differences in *LOX* expression could be the main reasons for the observed VOC changes between different storage-tolerant rice varieties after storage.

SOD and POD both prevent oxidative damage by removing reactive oxygen species (ROS) (Michalak et al., 2015). After storage, the activities of SOD and POD of all four rice varieties were significantly decreased compared to fresh rice (Fig. 5c and d). A previous study reported that high temperature and humidity storage conditions would decrease the gene expression of POD, in which conditions the POD activity would be reduced (Yuan et al., 2019). Decreased SOD and POD activities would lead to excessive accumulation of ROS, and the ROS would attack free fatty acids and participate LOX oxygenates unsaturated fatty acids, which have a negative impact on rice aroma (Liu et al., 2025). It was observed that SOD and POD activity in storage-tolerant varieties was higher than less storage-tolerant varieties in fresh and 6-month stored rice. Higher SOD and POD activities in N81 and JH1 were indicative of the metabolic status of rice and its adaptability to high temperature and humidity conditions during storage. This finding was similar to a previous study that presented evidence that the antioxidant enzyme activities of rice differed between rice varieties during storage (Zhu, Shao, et al., 2023).

### 3.5 Mechanism of flavor changes among different storage-tolerant rice varieties during storage

The change in rice flavor during storage was significant. Many previous studies focused on methods for reducing changes in rice flavor during storage. In general, there are two main strategies. The first is to reduce lipid oxidation by optimizing storage conditions, such as lowering the storage temperature (4 °C) or humidity (Zhao, Yousaf, et al., 2020). The other approach for reducing changes in rice flavor is to use physicochemical techniques to change the enzyme activities of paddy rice before storage. For example, dielectric barrier discharge cold plasma (DBD-CP) could promote the stabilization of brown rice and preserve flavor during storage (Liu, Wu, et al., 2021). This study reported genotypic differences in the accumulation of flavor-determining compounds during storage.

**Fig. 6:**
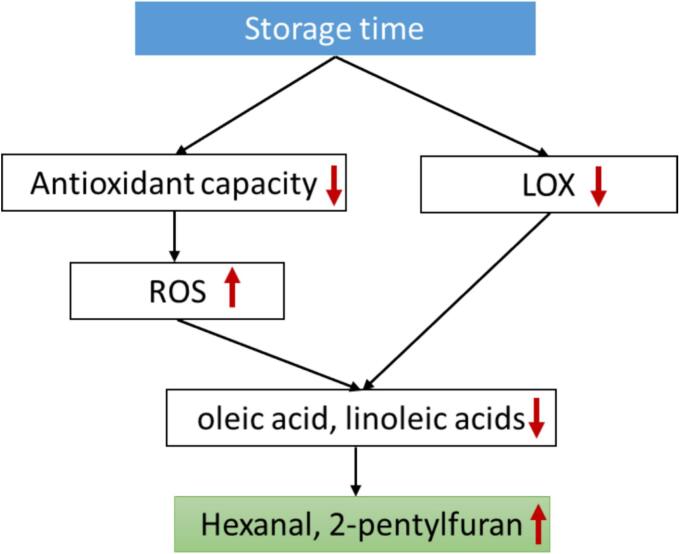
The possible model of lipid degradation and changes in rice flavor during rice storage.

We concluded that the changes in rice flavor during storage were mainly regulated by two pathways (Fig. 6). The most important one regulating rice flavor changes was the “LOX pathway,” in which storage time increased LOX activity, which hydrolyzed oleic and linoleic acids, etc. to produce hexanal and 2-pentylfuran which themselves negatively impact rice flavor. The second is the “oxidative stress pathway,” wherein the activities of antioxidant enzymes (POD and SOD) decreased during storage. Decreased antioxidant enzymes would cause excessive ROS accumulation in rice, accelerating the oxidation of unsaturated fatty acids. Similarly, it ultimately deteriorated the rice flavor. In this study, lower LOX activity in fresh rice and the second and fourth months of stored rice of N81 and JH1 due to their intrinsic genotypes could explain the changes in VOCs between the two groups of different storage-tolerant. This interesting finding suggests that downregulated LOX expression in seeds may reduce the activity of the LOX enzyme during storage, which might effectively reduce the oxidation of lipids and the production of unpleasant flavor during storage. This method may be more efficient and cost-effective than traditional physicochemical methods for maintaining rice flavor during storage.

## 4 Conclusion

This study investigated the changes in VOCs and physiological properties of different storage-tolerant rice varieties after storage. With the increasing storage time, LOX activity first increased and then decreased, while SOD and POD activities showed a significant decrease. Correspondingly, the oleic acid, linoleic acids, and sucrose content significantly decreased during storage. These changes in paddy rice cause changes in the VOCs of rice. Interestingly, the four *japonica* rice varieties during storage had particular patterns. Specifically, N81 and JH1 showed less change in VOCs during storage than N84 and ZJ96. This study reported for the first time the genotypic mechanisms underpinning the difference of flavor change in four *japonica* rice varieties during storage.

## CRediT authorship contribution statement

**Dawei Zhu:** Methodology, Formal analysis, Conceptualization. **Xin Zheng:** Writing – original draft, Investigation. **Huiyin Dong:** Methodology, Formal analysis. **Xingquan Liu:** Investigation, Supervision. **Xianqiao Hu:** Formal analysis. **Mingxue Chen:** Project administration, Formal analysis. **Xin Liu:** Project administration, Investigation. **Yafang Shao:** Supervision, Project administration.

## Declaration of competing interest

The authors declare that they have no known competing financial interests or personal relationships that could have appeared to influence the work reported in this paper.

## Data Availability

Data will be made available on request.

## A. Supplementary data

Supplementary tables and figures of rice quality and VOCs Image 1
