# Supplementary material for: Effects of storage on volatile organic components and physiological properties of different storage-tolerant rice varieties
Source: Food Chem X. 2024 Dec 27;25:102134. doi: 10.1016/j.fochx.2024.102134 (PMC11751419; doi:10.1016/j.fochx.2024.102134)
Supplement: Supplementary file 1 — Supplementary tables and figures of rice quality and VOCs [file mmc1.docx]

# Supplementary data

**Fig. S1**. Relative contents of different classes of volatile organic compounds of four rice varieties during storage.

 **Fig. S2**. Hexanal and 2-pentylfuran contents of four rice varieties.

**Table S1** Changes in rice quality stored at room temperature for one year

| Rice variety | Variety type | Fresh rice | | Stored rice | | Increase in fatty acid value | Decrease in eating quality |
| --- | --- | --- | --- | --- | --- | --- | --- |
|  |  | Fatty acid value | Eating quality | Fatty acid value | Eating quality |  |  |
| N84 | less storage-tolerant | 13.6a | 83.4a | 28.3a | 64.0b | 108.1 | -23.3 |
| ZJ96 |  | 14.6a | 84.0a | 30.0a | 60.5b | 105.5 | -28.0 |
| N81 | storage-tolerant | 12.3a | 82.5a | 21.2b | 70.3a | 72.4 | -14.8 |
| JH1 |  | 12.9a | 82.0a | 20.6b | 71.5a | 59.7 | -12.8 |

Values in the same column with different letters are significantly different (p < 0.05).

**Table S2** Volatile compound standard solution concentration

| Volatile compound | C_0_ (μg/mL) | C_1_ (μg/mL) | C_2_ (μg/mL) | C_3_ (μg/mL) | C_4_ (μg/mL) |
| --- | --- | --- | --- | --- | --- |
| Hexanal | 0.00 | 50.00 | 100.00 | 200.00 | 400.00 |
| 2-pentylfuran | 0.00 | 5.00 | 10.00 | 20.00 | 40.00 |

**Table S3** VOCs identified from four rice varieties

| Number | Volatile organic compounds | Retention index | Retention time (s) | Relative migration time (ms) |
| --- | --- | --- | --- | --- |
| 1 | Propyl hexanoate | 1108.6 | 821.957 | 1.38849 |
| 2 | 1-Nonanal (M) | 1101.5 | 803.198 | 1.47435 |
| 3 | 1-Nonanal (D) | 1101.1 | 802.156 | 1.94404 |
| 4 | Phenethyl alcohol | 1101.9 | 804.264 | 1.51847 |
| 5 | Diallyldisulfide | 1079.7 | 748.506 | 1.18766 |
| 6 | Acetophenone | 1065.1 | 714.124 | 1.18907 |
| 7 | (E)-2-Octenal | 1063.8 | 710.944 | 1.33031 |
| 8 | 2-Ethylhexanol | 1041 | 660.513 | 1.3894 |
| 9 | Dipentene | 1032.6 | 642.793 | 1.21501 |
| 10 | Octanal (M) | 1009.8 | 596.906 | 1.40669 |
| 11 | Octanal (D) | 1009.1 | 595.542 | 1.81454 |
| 12 | 2-Pentylfuran | 992.8 | 563.739 | 1.25248 |
| 13 | Benzaldehyde (M) | 977.9 | 531.986 | 1.14902 |
| 14 | Benzaldehyde (D) | 977.3 | 530.825 | 1.46464 |
| 15 | 1-Octen-3-ol | 991 | 559.815 | 1.17096 |
| 16 | (E)-2-Heptenal | 959.4 | 495.146 | 1.2544 |
| 17 | Heptaldehyde (M) | 902.4 | 397.052 | 1.33271 |
| 18 | Heptaldehyde (D) | 900.3 | 393.835 | 1.67968 |
| 19 | 2-Heptanone (M) | 897.2 | 389.011 | 1.26115 |
| 20 | 2-Heptanone (D) | 896.1 | 387.403 | 1.63783 |
| 21 | Hexyl alcohol (M) | 883.8 | 370.25 | 1.3219 |
| 22 | Hexyl alcohol (D) | 881.4 | 367.034 | 1.65133 |
| 23 | (E)-2-Hexenal (M) | 855.9 | 334.871 | 1.18162 |
| 24 | (E)-2-Hexenal (D) | 850.9 | 328.975 | 1.51344 |
| 25 | Hexanal (M) | 802 | 275.907 | 1.26531 |
| 26 | Hexanal (D) | 798.8 | 272.691 | 1.56043 |
| 27 | 2,3-Butanediol | 793.4 | 267.498 | 1.35768 |
| 28 | 1-Pentanol (M) | 778.9 | 253.516 | 1.24978 |
| 29 | 1-Pentanol (D) | 777.3 | 251.973 | 1.51101 |
| 30 | (E)-2-Pentenal | 764.8 | 240.249 | 1.12174 |
| 31 | Methyl 2-methylbutyrate | 766.2 | 241.483 | 1.19712 |
| 32 | 3-Methyl-1-butanol | 750.7 | 227.599 | 1.23739 |
| 33 | Valeraldehyde (M) | 700.5 | 187.797 | 1.19505 |
| 34 | Valeraldehyde (D) | 698.4 | 186.255 | 1.41911 |
| 35 | Isovaleraldehyde (M) | 660.4 | 166.2 | 1.1837 |
| 36 | Isovaleraldehyde (D) | 663.2 | 167.434 | 1.3995 |
| 37 | Methyl isobutyrate | 681.7 | 176.073 | 1.14136 |
| 38 | Ethyl acetate | 635 | 155.093 | 1.10419 |
| 39 | 2-Acetyl-1-pyrroline | 935.2 | 450.852 | 1.1245 |
| 40 | DL-2-Octanol | 1010.3 | 597.889 | 1.45576 |
| 41 | 1-Octen-3-one | 976.7 | 529.647 | 1.26525 |
| 42 | (E)-2-Heptenal (M) | 958.6 | 493.73 | 1.66236 |
| 43 | Methyl hexanoate (M) | 926.3 | 435.484 | 1.28785 |
| 44 | Methyl hexanoate (D) | 924.3 | 432.242 | 1.67735 |
| 45 | 2-Heptanol | 901.8 | 396.039 | 1.38455 |
| 46 | 2-Acetylfuran | 917.1 | 420.292 | 1.12556 |
| 47 | Phenylacetaldehyd | 1041.2 | 660.923 | 1.26174 |
| 48 | Hydroxyacetone (M) | 638.9 | 156.739 | 1.03903 |
| 49 | Hydroxyacetone (D) | 634.8 | 154.997 | 1.22521 |
| 50 | Triethylamine | 683.5 | 176.931 | 1.22606 |
| 51 | Propionic acid (M) | 721.5 | 203.504 | 1.11604 |
| 52 | (E)-2-Pentenal (D) | 766 | 241.315 | 1.37811 |
| 53 | 2-Butanone | 594.4 | 138.88 | 1.07604 |
| 54 | Acetic acid glacial | 625.8 | 151.255 | 1.07288 |
| 55 | Propionic acid (D) | 680.1 | 175.308 | 1.08599 |
| 56 | 1-Propanol (M) | 583.8 | 134.95 | 1.11758 |
| 57 | 1-Propanol (D) | 579.5 | 133.385 | 1.25448 |

(D), dimer, (M), monomer.
